# Characteristics of proteolytic microorganisms and their effects on proteolysis in total mixed ration silages of soybean curd residue

**DOI:** 10.5713/ajas.18.0933

**Published:** 2019-04-15

**Authors:** Wei Hao, Pengjiao Tian, Mingli Zheng, Huili Wang, Chuncheng Xu

**Affiliations:** 1 Department of Agricultural Engineering, College of Engineering, China Agricultural University, Beijing 100083, China; 2 State Key Laboratory of Feed Microbial Engineering, Beijing Da Bei Nong Science and Technology Group Co., Ltd., Beijing, China

**Keywords:** Aerobic Bacteria, Lactic Acid Bacteria, Proteolysis, Proteinase, Total Mixed Ration Silage

## Abstract

**Objective:**

The objective of this study was to isolate proteolytic microorganisms and evaluate their effects on proteolysis in total mixed ration (TMR) silages of soybean curd residue.

**Methods:**

TMRs were formulated with soybean curd residue, alfalfa or *Leymus chinensis* hay, corn meal, soybean meal, a vitamin-mineral supplement, and salt in a ratio of 25.0: 40.0:30.0:4.0:0.5:0.5, respectively, on a basis of dry matter. The microbial proteinases during ensiling were characterized, the dominate strains associated with proteolysis were identified, and their enzymatic characterization were evaluated in alfalfa (A-TMR) and *Leymus chinensis* (L-TMR) TMR silages containing soybean curd residue.

**Results:**

Both A-TMR and L-TMR silages were well preserved, with low pH and high lactic acid concentrations. The aerobic bacteria and yeast counts in both TMR silages decreased to about 10^5^ cfu/g fresh matter (FM) and below the detection limit, respectively. The lactic acid bacteria count increased to 10^9^ cfu/g FM. The total microbial proteinases activities reached their maximums during the early ensiling stage and then reduced in both TMR silages with fermentation prolonged. Metalloproteinase was the main proteinase when the total proteinases activities reached their maximums, and when ensiling terminated, metallo and serine proteinases played equally important parts in proteolysis in both TMR silages. Strains in the genera *Curtobacterium* and *Paenibacillus* were identified as the most dominant proteolytic bacteria in A-TMR and L-TMR, respectively, and both their proteinases were mainly with metalloproteinase characteristics. In the latter ensiling phase, *Enterococcus faecium* strains became the major sources of proteolytic enzymes in both TMR silages. Their proteinases were mainly of metallo and serine proteinases classes in this experiment.

**Conclusion:**

Proteolytic aerobic bacteria were substituted by proteolytic lactic acid bacteria during ensiling, and the microbial serine and metallo proteinases in these strains played leading roles in proteolysis in TMR silages.

## INTRODUCTION

There is a common practice in recent years to anaerobically ferment high-moisture and perishable agricultural and food by-products with dry feeds as total mixed ration (TMR) silages [1–3]. The efficient utilization of by-products helps to develop new feed resources for ruminants, and the ensiling process results in highly enhanced preservation [4], facilitating the transportation and flexible utilization of TMR without the occurrence of aerobic deterioration for a relatively long period [5].

Ensiling is a complex process that involves the interaction of plant enzymes and numerous microbial species, ultimately changing the biochemistry of silage [6]. Hydrolysis of protein is expected to occur during the whole fermentative stage, and it has been well accepted a result of the plant proteinases and the microbial activities in silages. Plant proteins can be degraded into oligopeptides, free amino acids, ammonia and other forms of non-protein nitrogen in silages (NPN) [7]. The utilization of silage NPN in rumen is often less efficient, and the inefficient NPN metabolism can lead to the nitrogen loss to the environment, which is now an increasing concern on silage-based diets in livestock production [8].

Proteolysis in ensiled forage has been mainly considered a result of the plant proteolytic enzymes [7,9]. So far, the plant proteinases and peptidases have been principally clarified, and their relative contributions to the formation of various NPN compounds have been well demonstrated in silages. According to Guo et al [10] and Tao et al [11], protein hydrolysis mainly resulted from the plant exo- and endopeptidases, and the principle exo- and endopeptidases hydrolyzing the forage protein were di-, tripeptidyl- and carboxypeptidases, and metallo and cysteine peptidases, respectively. McKersie and Buchanan-Smith [12] also demonstrated that carboxypeptidase, aminopeptidase, and acid proteinase were important in the degradative process of protein in ensiled alfalfa. In addition to the well-accepted effects of the plant proteinases, microbial activities were also reported to participate in the proteolysis during ensiling. According to Winters et al [13], microbial proteinases played an important role in the metabolism of amino acids in the ensiled ryegrass, and Heron et al [14] also pointed out that the ammonia and amines were the largely end products of microbial proteinases. Tao et al [15] investigated the effects of epiphytic bacteria and exogenous lactic acid bacteria (LAB) on the proteolysis in alfalfa silage, and concluded that both the aerobic bacteria and LAB species could affect the formation of NPN constitutes during the fermentative period. However, particular emphasis has been made in studies on the reactions involved in protein hydrolysis in regular silages, whereas the degradation of protein and its metabolism did not receive the same interest in TMR silages. In this experiment, we described the proteolytic enzymatic characterization and the dominant proteolytic bacteria succession in TMR silages during ensiling. Different classes of proteinases in the proteolytic isolates were also investigated in our study to elucidate the possible role of these specific strains in the hydrolysis of protein during anaerobic fermentation in TMR silages.

## MATERIALS AND METHODS

### Total mixed ration silages preparation and treatments

Alfalfa hay and *Leymus chinensis* hay were used for the A-TMR and L-TMR formulations in this study. As shown in Table 1, TMRs in this experiment were formulated with soybean curd residue, alfalfa or *Leymus chinensis* hay, corn meal, soybean meal, a vitamin-mineral supplement, and salt in a ratio of 25.0:40.0:30.0:4.0:0.5:0.5, respectively, on a basis of dry matter (DM). The soybean curd residue was obtained from a local food factory in Beijing and used within 2 h of production, and the hays were chopped to a length of 1 to 2 cm prior to mixing. Approximately 200 g well-mixed TMRs were filled into plastic film bags (Hiryu KN type, 200×300 mm; Asahikasei, Tokyo, Japan), degassed and sealed using a vacuum packing machine (BH950, Matsushita, Osaka, Japan). The experiment was carried out in a completely randomized design. Total twenty-seven bags per treatment were stored in a room with temperature maintained at 22°C to 25°C and triplicate bags were randomly opened at 0, 1, 3, 5, 7, 10, 14, 28, and 56 d of ensiling for laboratory analysis.

### Chemical analysis

To determine the nutritional value of the soybean curd residue and TMRs, DM, organic matter, and crude protein were determined by the methods of 934.01, 942.05, and 976.0, respectively, of AOAC [16], acid detergent fiber and neutral detergent fiber were analyzed as described by Van Soest et al [17], and water soluble carbohydrate was measured using the method of McDonald and Henderson [18]. Fermentation quality of TMR silages was determined by cold water extracts. Samples (10 g) randomly collected were homogenized with 90 mL autoclaved sterilized distilled water at 4°C and then filtered through four layers of cheesecloth. The filtrates were stored at −80°C until analysis for pH, ammonia nitrogen (NH_3_-N), free amino acid nitrogen (FAA-N) and organic acids. pH was measured with a glass electrode pH meter (Mettler Toledo S20, Greifensee, Switzerland) and the concentrations of lactic, acetic, propionic and butyric acids were measured by high performance liquid chromatography (LC-10A, Shimadzu, Tokyo, Japan) as described by Xu et al [1]. The NPN concentration was calculated by the method of Licitra et al [19]. To determine the composition of NPN fractions, an aliquot of 5 mL (250 g/L) trichloroacetic acid (TCA) was added to 20 mL of the filtrate to precipitate protein, and after centrifugation (12,000 *g*, 15 min, 4°C), the supernatant was analyzed for NH_3_-N and FAA-N by the method of Broderick and Kang [20]. Peptide nitrogen (peptide-N) was determined by the increase in FAA-N in the TCA supernatant after digesting with 6 mol/L of HCl for 21 h at 105°C under an N_2_ atmosphere.

### Crude enzyme extraction and determination

For analysis of the microbial proteinase activity in TMRs and TMR silages, a second sample, weighing approximately 10 g, was homogenized in 40 mL sterilized distilled water. The 10,000 *g* supernatant served as the crude extract for the analysis of proteolytic enzymes.

Proteinase activity against azocasein as the substrate was determined as described by Fischer et al [21] with some slight modifications. In this experiment, 0.5 mL of the each enzyme extract was mixed with 0.5 mL 10 g/L azocasein in 0.2 mmol/L pH 5.5 Na-acetate buffer, and the mixture was incubated for exactly 3 h at 37°C. Undigested azocasein was precipitated by adding 0.5 mL 150 g/L cold TCA, and then let stand before centrifugation (12,000 *g*, 10 min, 4°C). One unit of proteinase activity was equivalent to the increase of 0.01 absorbance unit of supernatant at 440 nm per hour.

Proteinases are divided into four main classes based on their action sites on protein, namely serine, cysteine, aspartic, and metallo proteinases [22]. One specific inhibitor of the above proteinases was added to the crude enzyme extracts to determine the classes and relative activities of the microbial proteinases in TMR silages. Treatments included a serine proteinase inhibitor (phenylmethylsulfonyl fluoride, PMSF), an aspartic proteinase inhibitor (pepstatin A), a metalloproteinase inhibitor (1,10-phenanthroline), and a cysteine proteinase inhibitor (E-64) [10]. All inhibitors were purchased from Sigma Chemical Co., Poole, UK. After dissolving the inhibitors in dimethyl sulfoxide (DMSO), triplicate samples of crude extracts were incubated with one specific inhibitor at 22°C for 30 min before determination of proteinase activity. Final concentrations were 10 mmol/L of PMSF, 1 mmol/L of pepstatin A, 10 mmol/L of 1,10-phenanthroline, and 1 mmol/L of E-64, respectively, in order to suppress the corresponding proteinase as completely as possible. Controls were prepared with DMSO, but without addition of specific proteinase inhibitors.

### Isolation of proteolytic bacteria and measurement of proteolytic activity

Populations of the proteolytic microorganisms were measured through the spread-plate count method. Two different media were used for the isolation of proteolytic bacteria. Medium 1 was modified nutrient agar with 10 g/L of peptone replaced by 5 g/L of casein for proteolytic strains of aerobic bacteria [23], and medium 2 was the modified MRS (de Man Rogosa Sharpe) agar with 5 g/L of casein as the single nitrogen source for the isolation of proteolytic LAB strains. Samples (10 g) of TMRs and TMR silages were homogenized in 90 mL sterilized distilled water, and were 10^−1^ to 10^−5^ serially diluted. Portions (100 μL) of dilutions were spread on the modified media. After cultivation under recommended conditions, colonies on these agar media that produced transparent circles were picked, purified, and then were conserved in 20% glycerol at −80°C for further analysis. To identify the species of the purified isolates, polymerase chain reaction (PCR) was carried out to amplify the complete 16S rRNA gene sequence with the forward primer 27f (5′-AG AGTTTGATCCTGGCTCAG- 3′) and the reverse primer 1492r (5′-GGTTACCTTGTTACG ACTT-3′). The PCR procedure was performed as described by Hu et al [3]. The PCR products were separated by gel electrophoresis on a 1.0% agarose gel, detected by Gold View (Solarbio, Beijing, China) according to the manufacturer’s instructions and photographed under UV light with a charge-coupled device camera. Sequencing was carried out by Shanghai Sunny Biotechnology Co., Ltd. (Shanghai, China), and then the sequences were analyzed using BLASTN online tool (http:/blast.ncbi.nlm.nih.gov/Blast.cgi).

The isolates were inoculated into the corresponding liquid form of the isolation media to determine the activities and compositions of the proteinases produced by the dominant proteolytic strains. When these cultures had reached stationary phase (usually after 24 h, but at earlier times for LAB), the cells were pelleted by centrifugation at 6,000 *g* for 10 min at 4°C, and the samples of the supernatant were used for the determination of proteinase activity. The extent of azocasein digestion was estimated as described previously, with following modifications: 0.5 mL of the each supernatant was used as the crude extract of microbial proteinase, and the incubation time was switched to 0.5 h. One unit of microbial proteinase activity was described as increase of 0.01 absorbance unit at 440 nm per hour.

The partial 16S rRNA gene sequences were deposited in the GenBank database under the accession nos. KC412560–KC412633.

### Statistical analysis

Statistical analysis was performed using the general linear model procedure of IBM SPSS Statistics for Windows (Version 20.0; IBM Co., Armonk, NY, USA). Data on the fermentation products, NPN constitutes, and microbial proteinase activities in A-TMR and L-TMR silages were subjected to two-way analysis of variance, with the fixed effects of days of ensiling, type of TMR silages, and the interactions between days of ensiling and type of TMR silages. Significance was defined at a 0.05 probability level.

## RESULTS

### Chemical compositions of total mixed rations and total mixed ration silages

Initial nutritional values of TMRs and soybean curd residue are shown in Table 1.

Table 2 shows the dynamic changes of fermentation qualities in A-TMR and L-TMR silages. Ensiling period significantly decreased the pH values in TMR silages (p<0.001), and after 56 d of ensiling, pH values declined to 4.22 and 4.21 in A-TMR and L-TMR silages, respectively. Both lactic and acetic acids concentrations increased with the prolonged ensiling days (p<0.001), and A-TMR exhibited higher contents of lactic and acetic acids than L-TMR silage throughout the ensiling period (p<0.05). Butyric acid was not detected in any of the TMR silages of this experiment.

### Characteristics of proteolysis in total mixed ration silages

As shown in Table 3, proteolysis in both TMR silages was extensive, and the NPN contents increased to approximately 335 and 310 g/kg of the total nitrogen (TN) in 56 d A-TMR (p<0.001) and L-TMR (p<0.001) silages, respectively. Proportions of peptide-N and FAA-N significantly increased with the prolonged ensiling period (p<0.001), and peptide-N accounted for the largest of the NPN fractions. Concentration of peptide-N was also significantly affected by the type of TMR silage (p<0.001) and the interaction between the ensiling time and the TMR silage type (p<0.05), while, there was no significant difference in FAA-N concentration between A-TMR and L-TMR silages during ensiling period (p>0.05). NH_3_-N concentrations significantly increased in both TMR silages with the increased fermentative duration (p<0.001), and when ensiling terminated after 56 d, L-TMR silage was characterized by a significantly higher NH_3_-N concentration than A-TMR silage in this experiment (p<0.001).

### Proteinase activities in total mixed ration silages at different stages of ensiling

**Figure 1:**
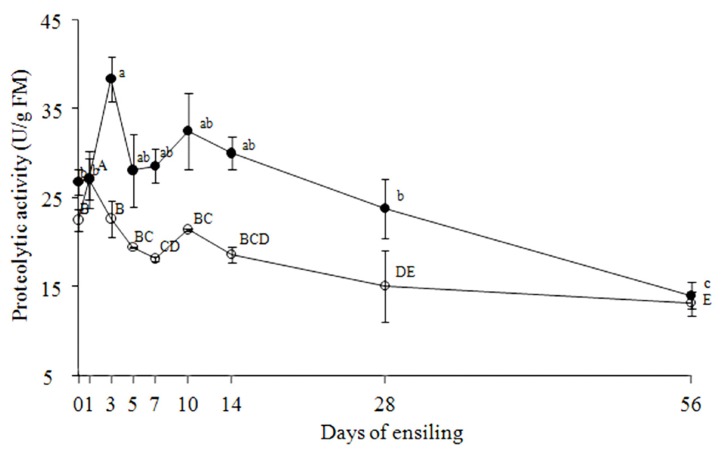
Proteolytic activities in A-TMR (○) and L-TMR (●) silages at different stages of ensiling. A-TMR, total mixed ration formulated with alfalfa hay; L-TMR, total mixed ration formulated with Leymus chinensis hay. One unit of proteinase activity was equivalent to the increase of 0.01 absorbance unit of the supernatant at 440 nm per hour. Data not sharing a common letter differ (p<0.05). The lowercase letters identify the significance of the data in L-TMR silage, and the capital letters identify the significance of the data in A-TMR silage.

Figure 1 exhibits the dynamic changes of the proteinases activities in TMR silages during ensiling, and the activities of the total microbial proteinses based on the description of the method were significantly affected by the ensiling period (p< 0.001), the type of TMR silages (p<0.001), and their interaction (p<0.001) in this experiment. In fresh, unensiled L-TMR and A-TMR the activities of the proteinases against azocasein were equivalent to 26.3 U/g and 22.0 U/g, respectively. In L-TMR, the proteolytic activity increased to its maximum of 37.8 U/g fresh matter (FM) at 3 d of ensiling, and thereafter, the activity was reduced to 13.6 U/g FM at 56 d (p<0.001). The pattern in A-TMR silage was similar with that in L-TMR silage. The proteolytic activity reached a lower peak of 26.6 U/g FM at 1 d, and were subsequently decreased to approximately 12.7 U/g FM at 56 d of ensiling (p<0.001).

**Figure 2:**
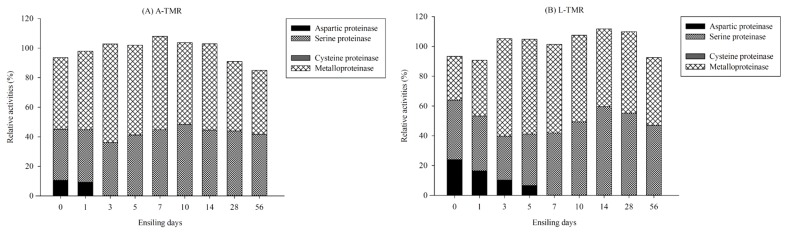
Relative activities of aspartic, serine, cysteine and metallo proteinases in A-TMR and L-TMR silages at different stages of ensiling. A-TMR, total mixed ration formulated with alfalfa hay; L-TMR, total mixed ration formulated with Leymus chinensis hay.

The relative activities of different classes of proteinases in both TMR silages are shown in Figure 2. In the unensiled A-TMR, the portions of aspartic, serine, and metalloproteinase were approximately 10.5%, 34.5%, and 48.5% of the total microbial proteinase based on the description of the method, respectively. When ensiling process was prolonged to 3 d, the relative activity of metalloproteinase was increased to 66.7%, while aspartic proteinase was absent. In L-TMR silage, the initial aspartic proteinase accounted for approximately 23.9% of the total microbial proteinase, and then its relative activity progressively decreased until it was inactive at 7 d. The relative activity of metalloproteinase increased to 65.6% by 3 d and then dropped gradually along with the fermentation process. At the end of the ensiling, metallo and serine proteinases both played important parts in the hydrolysis of protein. There was no cysteine proteinase detected in A-TMR and L-TMR silages throughout the whole fermentative stages.

### Identification of proteolytic bacteria in total mixed ration silages

In A-TMR and L-TMR silages, respectively, 488 and 583 strains were isolated during the ensiling period, and all the isolates were evaluated for their ability to secrete proteinases. Of these isolates, 102 and 88 were predominantly aerobic bacteria and *Bacillus* strains in A-TMR silage, respectively, and in L-TMR silage, 67 and 176 strains were identified as proteolytic aerobic bacteria and *Bacillus* strains, respectively. The rest of the isolates were LAB strains positive for proteinase secretion when grown on the casein agars.

The successions of dominant proteolytic bacteria isolated in TMR silages are shown in Table 4. In A-TMR silage, proteolytic strains of *Curtobacterium flaccumfaciens* (*C. flaccumfaciens*) and *Curtobacterium citreum* (C*. citreum*)were predominant during the initial fermentation stage. By 3 d, LAB occurred in a larger population, and the positive strains were identified as *Lactobacillus plantarum (L. plantarum*), *Pediococcus acidilactici* (*P. acidilactici*) and *Enterococcus faecium* (*E. faecium*). Proteolytic strains of *Enterococcus mundtii* (*E. mundtii*) were only discovered at 7 d and 10 d in A-TMR silage. When ensiling period prolonged, the relative abundance of *P. acidilactici* gradually decreased, and by 28 d, the main sources of proteolytic enzymes in A-TMR silage were the proteolytic strains of *E. faecium* and *L. plantarum*. In L-TMR silage, *Paenibacillus borealis (P. borealis*), *Paenibacillus turicensis* (*P. turicensis*) and *Paenibacillus xylanexedens* (*P. xylanexedens*) were determined as dominant proteolytic bacteria during the first 3 d of ensiling. By 5 d, *L. plantarum*, *E. faecium* and *P. acidilactici* occurred predominantly. As fermentation prolonged, proteolytic *P. acidilactici* lost its predominant position. *E. faecium* and *L. plantarum* were inhibited as well, but still a main source of microbial proteinase during the latter phase of ensiling. Proteolytic *Bacillus* strains could be isolated in lower order of magnitudes throughout the whole ensiling period, and these strains were identified as *Bacillus safensis*, *Bacillus flexus*, *Bacillus cereus*, *Bacillus amyloliquefaciens* (*B. amyloliquefaciens*) and *Bacillus methylotrophicus* (*B. methylotrophicus*) in both TMR silages.

### Characterization of proteinases produced by dominant proteolytic bacteria in total mixed ration silages

Table 5 exhibits the activities and characteristics of the proteinases produced by the dominant proteolytic bacteria in both TMR silages. Activities of the proteinases in *C. citreum* and *C. flaccumfaciens* were equivalent to 3.2 to 4.7 U/mL and 6.0 to 6.6 U/mL, respectively, and both the proteinases could be strongly suppressed by 1,10-phenanthroline, showing the characteristics of metalloproteinase. The proteolytic activities of *B. amyloliquefaciens* and *B. methylotrophicus* were extremely high. Their proteinases were largely suppressed by 1,10-phenanthroline and PMSF, showing their proteinases were mainly a serine-metallo proteinase type. The proteinases in other isolated species of genus *Bacillus* mainly had serine proteinase characteristics, and exhibited aspartic and metallo proteinases activities. In *P. borealis*, *P. xylanexedens* and *P. turicensis*, the activities of extracellular proteinases were approximately 5.6 to 5.8 U/mL, 9.2 to 9.6 U/mL, and 5.7 to 6.4 U/mL, respectively. Their proteinases were mainly metalloproteinase, and also with aspartic proteinase in their proteolytic systems. *L. plantarum*, *E. faecium*, and *P. acidilactici* presented relatively low proteolytic activities. Proteinase in *L. plantarum* were strongly suppressed by PMSF, and the *P. acidilactici* proteinase were more sensitive to 1,10-phenanthroline. In *E. faecium* and *E. mundtii*, the extracellular proteinases were strongly suppressed by 1,10-phenanthroline and also inhibited by PMSF in a relatively lower level, exhibiting metallo and serine proteinases characteristics in this experiment.

## DISCUSSION

One of the most significant processes occurring during ensiling is the enzymatic degradation of protein to the NPN forms in silages. Even in well-preserved silages, approximately 50% degradation of protein may take place [24,25]. Alfalfa is particularly susceptible to proteolysis, because the proteinase activity is higher in this crop than in other grasses or legumes [26]. So far, studies have been mainly focused on the proteolysis in silages, especially in alfalfa silage, while the degradation of protein and its mechanism has not been well demonstrated in TMR silages. This experiment showed the intensities of proteolysis, activities and characterizations of proteinases, and the dominant proteolytic microorganisms in A-TMR and L-TMR silages. Furthermore, it was the first attempt to determine the contributions of the predominating proteolytic species to the hydrolysis of protein in TMR silages, and might aid to elucidate the mechanism of proteolysis from a microbial perspective.

Both plant enzyme and microbial activity are factors responsible for the extensive breakdown of protein in silages. It has been well accepted that the initial hydrolysis is mediated by plant enzymes, and the subsequent transformation of amino acids to ammonia and amines is mostly brought about by the activities of proteolytic microorganisms [7]. However, the above statement that proteolytic activity during ensiling is largely attributed to plant enzymes seems not to be entirely suitable for TMR silages in this experiment. Soybean curd residue and other food by-products, such as brewers’ grains [1], green tea ground, and apple pomace [2] which are commonly utilized to replace the commercial feedstuffs in TMRs might have lost their enzymatic activities through the procedures of food processing and manufacturing, and the enzymes in dry feeds or hays might also be inactive because of their low levels of the moisture contents. Thus, in this experiment, it could be assumed that the breakdown of protein in TMR silages was predominantly a microbial process.

Total activities of the microbial proteinases in both TMR silages exhibited dynamic changes during the ensiling process. Proteinases produced by microorganisms are complex and their activities can be affected by many factors, such as microorganism strain, substrate, temperature, and pH value. In this experiment, the increases of the proteinase activities during initial ensiling stage could be largely due to the temporary proliferation of the proteolytic species of aerobic bacteria, and the following decreases might be related to the inhibition of aerobic bacteria and the multiplication of LAB under the anaerobic and slightly acidic environments. Tao et al [15] once reported the effects of epiphytic microorganisms and exogenous LAB on the extent of proteolysis in alfalfa silages. Control silage had significantly higher concentrations of peptide-N, FAA-N and NH_3_-N than the silage inoculated with exogenous LAB after UV radiation, and this result roughly indicated that the epiphytic aerobic bacteria largely contributed to the formation of NPN compositions during ensiling period. Except for the increased population of the proteolytic LAB strains and their relatively low proteinase activities, the predominantly reduced pH value might also be an important factor leading to the inhibited proteolytic activities in TMR silages when ensiling prolonged. McKersie and Buchanan-Smith [27] reported that the activity of the native proteinase against azocasein (pH 5.5) decreased with the reduced pH value in alfalfa silage, and the proteinase activity retained only 30% of its original activity at 21 d after the anaerobic fermentation.

Proteinase can be classified according to the active amino acid residues at the enzyme reaction point, namely, aspartic, serine, cysteine and metallo proteinases, and different classes of proteinases make different contributions to the hydrolysis of protein. According to Guo et al [10], aspartic and cysteine proteinases mainly contribute to the degradation of protein into oligopeptides, serine and metallo proteinases principally hydrolyze peptides into free amino acids, and the formation of NH_3_-N is largely due to metalloproteinase in alfalfa silages. In this experiment, the above four classes of proteinases were determined with the application of their specific inhibitors. Results showed that the serine and metallo proteinases accounted for the majority of the microbial proteinases in both TMR silages, and L-TMR silage exhibited significantly higher values of the absolute activities of serine and metallo proteinases than A-TMR silage throughout the whole ensiling period. This was consistent with the result that L-TMR silage obtained higher intensities of the degradation of peptides and higher concentrations of free amino acids and NH_3_-N during ensiling in our study. The microbial aspartic and cysteine proteinases were either significantly suppressed during the initial fermentation stage or totally inactive throughout the whole ensiling period, and this might be a reason that TMR silages exhibited significantly lower degrees of protein breakdown than regular silages, and the gradually decreased activities of the aspartic proteinases in both TMR silages might lead to the phenomenon that the rising rates of the peptide concentrations progressively declined with the increased fermentative duration. In addition, the initial hydrolysis of protein and the subsequent degradation of peptides into free amino acids might also result from the exopeptidases activities in TMR silages. According to Tao et al [11], the endo- and exopeptidases both contribute to the protein hydrolysis and the tripeptidyl-peptidase was a principle exopeptidase for protein degradation in alfalfa silage. So, there is also a necessity for another determination of the microbial exopeptidases in TMR silages in a further study to conclusively link the observed proteolysis with the specific bacteria species which have the capability of producing exopeptidases during the ensiling process.

Casein was used as the single nitrogen source in the selective media for the isolation of proteolytic strains in our study. However, it has been confirmed that these selective media are not particularly efficient for selecting proteolytic microorganisms [28], and a number of proteolytic isolates might fail to clear the casein-containing medium, presumably because their cell-bound proteinase cannot diffuse through the medium. So in this experiment, strains without clear haloes were also isolated and purified for their further analysis of the proteinase-producing abilities. In the unensiled A-TMR, proteolytic strains of *C. flaccumfaciens* and *C. Citreum* were isolated from 10^−3^ dilutions of the leaching solutions, and these strains were supposed to be the most dominant proteolytic isolates during the initial fermentation stage. The proteinases in *C. flaccumfaciens* and *C. Citreum* were mainly of the metalloproteinase type and this was in accord with the result that metalloproteinase occupied most of the total microbial proteinases in the initial A-TMR silage. In the unensiled L-TMR, *P. borealis*, *P. turicensis*, and *P. xylanexedens* were isolated as the most dominant proteolytic strains in this experiment. Their proteinases mainly had metalloproteinase characteristics, and aspartic proteinase was also weakly active in their proteolytic systems. In our study, the total microbial proteinase reached its maximal activity at 3 d, with metalloproteinase taking the majority of the proteinases in L-TMR silage, and these results were consistent with the dynamics of the populations of the proteolytic *Paenibacillus* strains during the initial fermentation. The relative activity of the aspartic proteinase gradually decreased even with the proliferation of *Paenibacillus* strains in L-TMR silage, this might be a result from the significantly reduced pH values, since the aspartic proteinase is a neutral protease, and might be totally inactive at pH 5 [9]. There are very limited reports about the proteolytic activity expressed by members of the genus *Paenibacillus*. Hang et al [29] isolated a proteolytic strain of *Paenibacillus* spp. from raw yak milk, and discovered that the enzyme activity was completely inhibited by ethylenediaminetetraacetic acid, indicating it was a metalloproteinase. The extracellular proteinase profiles of the other two *Paenibacillus* species, *Paenibacillus peoriae* and *Paenibacillus polymyxa* were also analyzed. The results showed that both the proteinases were at neutral-alkaline pH range, and of metalloproteinase class [30]. *B. amyloliquefaciens* and *B. methylotrophicus* were the most active isolates in both TMR silages of this experiment. Their activities were mainly with serine and metallo proteinases characteristics, and similar results were also obtained by Sai-Ut et al [31] before. Proteolytic strains of other *Bacillus* species were isolated in lower orders of magnitudes. Since *Bacillus* species are well known for their capability of producing the extracellular alkaline proteinases, with their activities remarkably decreased at the acidic pH range, the contributions of the genus *Bacillus* to proteoylsis could be definitely weakened with the increased fermentative duration.

Since LAB have a complex proteolytic system capable of converting protein to free amino acids [32], the proteolytic activity during the later ensiling phase could be due to the proteolytic strains of the genus *Lactobacillus* in TMR silages. Highly proteolytic strains of *Lactobacillus helveticus*, *Lactobacillus paracasei* (*L. paracasei*) subsp. *paracasei*, *Lactobacillus acidophilus*, *Lactobacillus casei (L. casei*), *Lactobacillus buchneri* (*L. buchneri*), and *Lactobacillus delbrueki* subsp. *bulgaricus* have been identified previously [33]. So far, many studies have characterized the amino acids changes mediated by LAB in the mature silages, such as the metabolisation of arginine by strains of *E. faecium*, *Enterococcus faecalis*, *L. buchneri*, *L. plantarum*, and *L. casei* [34], and the fermentation of serine and proline, respectively, by strains of *L. plantarum* and *L. paracasei* [13]. There are few studies examining the effects of LAB proteinases to the initial hydrolysis of protein to peptides in TMR silages. *L. plantarum*, *P. acidilactici*, and *E. faecium* were isolated as the proteolytic LAB strains in our study. Proteinase activities in proteolytic strains of *L. plantarum* are relatively low, and the inhibitor study suggested that the *L. plantarum* proteolytic enzyme was a serine proteinase. The proteolytic activities in *L. plantarum* have been reported helping in casein degradation in milk [35], and the characterization of serine proteinase were also reported in a *L. plantarum* proteinase by Hegazi and Abo-Elnaga [36]. The proteinase produced by a proteolytic strain of *P. acidilactici* has been studied previously. The results showed that the proteinase was an acidic proteinase [37], and this confirmed the result that *P. acidilactici* contributed to the proteolysis in both mature TMR silages with pH values of approximately 4.2 in this experiment. Proteoyltic activities of genus *Enterococci* were mainly described in milk products. The maximal degradation of milk protein was achieved at pH 6.5 and at 42°C for *E. faecium*, and the proteinase largely exhibited metalloproteinase characteristics according to El-Ghaisha et al [38]. In our study, the proteinases in the determined *E. faecium* strains mostly belonged to the metalloproteinase class, and serine proteinase was also active in their proteolytic systems. These results were mainly inconsistent with previous studies, and the difference between the results may be due to the different proteolytic strains of *E. faecium* in our study relative to the previous reported researches. Our study indicated that, with increased fermentative duration, the proteolytic aerobic bacteria strains were substituted by the proteolytic LAB, and the serine and metallo proteinases in these proteolytic strains played leading roles in the hydrolysis of protein in TMR silages. This work throws new light on the mechanisms of protein hydrolysis occurring in TMR silage from a microbial perspective, and further studies are needed to isolate the strains with exopeptidase activities and to evaluate the effects of these proteolytic species on the formation of NPN in TMR silages. There is also necessity to establish the significance of the endo- and exo- peptidases inhibitors on the protein quality in TMR silages, in the hope that it can provide some useful information for commercial development of proteolysis-inhibiting additives for TMR silages.

## Figures and Tables

**Table 1:** Ingredient proportions and chemical composition of total mixed rations and soybean curd residue

Items	Soybean curd residue	A-TMR	L-TMR
Ingredient (g/kg DM)			
Soybean curd residue	-	250	250
Alfalfa hay	-	400	0
*Leymus chinensis* hay	-	0	400
Corn meal	-	300	300
Soybean meal	-	40	40
VMS1)	-	5	5
Salt	-	5	5
Chemical composition (g/kg DM)			
DM (g/kg FM)	180±1.2	489±5.2	498±3.7
OM	968±10.7	930±9.2	929±8.1
CP	277±1.4	168±3.1	156±3.2
aNDF	320±8.5	303±11.4	342±8.6
ADF	195±10.3	202±5.7	205±8.2
WSC	169±3.2	146±8.8	151±4.6

Data of chemical composition are presented as mean±SD of 3 replicates.

A-TMR, total mixed ration formulated with alfalfa hay; L-TMR, total mixed ration formulated with *Leymus chinensis* hay; DM, dry matter; FM, fresh matter; OM, organic matter; CP, crude protein; aNDF, neutral detergent fiber assayed with a heat-stable amylase; ADF, acid detergent fiber; WSC, water soluble carbohydrate.

1) VMS, vitamin-mineral supplement is from commercial product (Longde feed, Hebei, China) containing 12 g/kg Zn, 10 g/kg Mn, 5 g/kg Fe, 2 g/kg Cu, a minimum of 5,000 IU of vitamin A/g, 600 IU of vitamin D/g.

**Table 2:** Dynamic changes in fermentation quality in A-TMR and L-TMR silages during ensiling

Items		Ensiling time (d)									SEM	p-value		
	
		0	1	3	5	7	10	14	28	56		D	T	T×D
pH	A-TMR	6.58^a^	5.80^bc^	5.64^c^	4.81^d^	4.61^de^	4.52^e^	4.25^f^	4.23^f^	4.22^f^	0.013	**	**	**
	L-TMR	6.77^a^	6.51^a^	5.82^b^	5.37^c^	4.98^d^	4.63^e^	4.43^ef^	4.38^ef^	4.21^f^				
Lactic acid	A-TMR	8.5^g^	12.2^f^	32.7^e^	44.3^d^	50.4^cd^	55.9^c^	66.3^b^	67.8^b^	72.5^a^	0.97	**	*	**
(g/kg DM)	L-TMR	7.2^f^	7.6^f^	11.4^e^	26.4^d^	48.8^c^	55.7^b^	59.4^b^	66.6^a^	68.1^a^				
Acetic acid	A-TMR	4.1^d^	5.8^d^	7.8^cd^	8.6^c^	9.7^b^	10.6^b^	11.2^b^	11.4^b^	12.7^a^	0.15	**	**	NS
(g/kg DM)	L-TMR	2.9^f^	3.5^e^	7.7^d^	8.3^c^	10.2^b^	10.2^b^	10.2^b^	10.7^ab^	10.9^a^				
Propionic acid	A-TMR	0.4^f^	2.2^e^	2.2^e^	3.0^d^	3.4^d^	4.6^c^	5.6^b^	7.9^a^	8.5^a^	0.21	**	NS	NS
(g/kg DM)	L-TMR	0.5^f^	3.7^d^	2.7^e^	2.9^e^	3.5^d^	4.4^c^	5.2^b^	6.0^a^	5.3^ab^				
Butyric acid	A-TMR	ND	ND	ND	ND	ND	ND	ND	ND	ND	-	-	-	-
(g/kg DM)	L-TMR	ND	ND	ND	ND	ND	ND	ND	ND	ND				

Data are presented as means of three replicates.

A-TMR, total mixed ration formulated with alfalfa hay; L-TMR, total mixed ration formulated with *Leymus chinensis* hay; SEM, standard error of mean; D, effect of days of ensiling; T, effect of type of TMR; D×T, interaction between days of ensiling and type of TMR; NS, not significant; ND, not detected.

a–g Means within rows not sharing a common letter differ (** p<0.001; * p<0.05).

**Table 3:** Dynamic changes of proportions of total N in NPN fractions in A-TMR and L-TMR silages during ensiling

Items		Ensiling time (d)									SEM	p-value		
		
		0	1	3	5	7	10	14	28	56		D	T	T×D
NPN	A-TMR	148.5^g^	177.3^f^	209.0^e^	228.7^de^	239.5^cd^	253.6^c^	276.5^b^	317.4^a^	335.0^a^	1.68	**	**	NS
(g/kg TN)	L-TMR	110.0^h^	127.0^g^	154.6^f^	178.9^e^	194.7^de^	207.3^d^	230.6^c^	282.8^b^	309.7^a^				
Peptide-N	A-TMR	119.7^e^	138.0^de^	159.9^cd^	168.6^bc^	181.4abc	186.1^ab^	190.0^ab^	192.2^a^	196.1^a^	1.05	**	**	*
(g/kg TN)	L-TMR	94.5^d^	98.2^d^	108.9^cd^	114.2^cd^	119.5^bc^	121.1^bc^	134.4^ab^	142.0^a^	147.1^a^				
FAA-N	A-TMR	24.7^g^	33.3^f^	37.3^ef^	36.5^ef^	43.0^e^	50.3^d^	64.7^c^	95.8^b^	107.1^a^	0.28	**	NS	**
(g/kg TN)	L-TMR	12.7^i^	23.8^h^	30.5^g^	41.5^f^	49.1^e^	58.9^d^	65.4^c^	96.5^b^	109.4^a^				
NH_3_-N	A-TMR	4.0^g^	5.9^g^	11.7^f^	13.6^ef^	15.0^e^	17.1^d^	21.8^c^	29.4^b^	31.8^a^	0.25	**	**	**
(g/kg TN)	L-TMR	2.8^g^	5.0^g^	15.2^f^	23.3^e^	26.2^d^	27.3^d^	30.8^c^	44.4^b^	53.2^a^				

Data are presented as means of three replicates.

NPN, non-protein nitrogen; A-TMR, total mixed ration formulated with alfalfa hay; L-TMR, total mixed ration formulated with *Leymus chinensis* hay; SEM, standard error of mean; D, effect of days of ensiling; T, effect of type of TMR; D×T, interaction between days of ensiling and type of TMR; FAA-N, free amino acid nitrogen; TN, total nitrogen; NH_3_-N, ammonia nitrogen; NS, not significant.

a–i Means within rows not sharing a common letter differ (** p<0.001; * p<0.05).

**Table 4:** Community successions of dominant proteolytic bacteria in A-TMR and L-TMR silages during fermentation stage

Items	Most closely type strain / accession no. (% similarity)	Populations of the dominant proteolytic bacteria (cfu/g FM)								
		0	1	3	5	7	10	14	28	56
A-TMR	*Curtobacterium flaccumfaciens* LMG 3645^T^ / AJ312209 (99%–100%)	3.3×10^5^	3×10^5^	ND	ND	ND	ND	ND	ND	ND
	*Curtobacterium citreum* DSM 20528^T^ / AM410690 (99%)	2.8×10^5^	1.1×10^5^	ND	ND	ND	ND	ND	ND	ND
	*Bacillus safensis* NBRC 100820^T^ / AB681259 (99%–100%)	3×10^3^	3×10^3^	1×10^2^	6×10^2^	4×10^2^	1×10^3^	2×10^2^	2×10^2^	2×10^2^
	*Bacillus flexus* NBRC 15715^T^ / AB680944 (99%–100%)	3×10^3^	2×10^3^	3×10^2^	2×10^2^	5×10^2^	4×10^2^	2×10^2^	2×10^2^	3×10^2^
	*Bacillus cereus* NBRC 15305^T^ / AB271745 (99%–100%)	5×10^2^	1×10^3^	3×10^2^	2×10^2^	2×10^2^	5×10^2^	3×10^2^	3×10^2^	ND
	*Bacillus amyloliquefaciens* NBRC 15535^T^ / AB255669 (99%)	3×10^2^	2×10^2^	3×10^2^	ND	ND	ND	ND	ND	ND
	*Bacillus methylotrophicus* CBMB 205^T^ / EU194897 (99%)	3×10^2^	3×10^2^	ND	ND	ND	ND	ND	ND	ND
	*Lactobacillus plantarum* NBRC 15891^T^ / AB326351 (99%–100%)	ND	ND	5.1×10^6^	5.4×10^7^	1.8×10^6^	1.7×10^6^	2×10^6^	2×10^4^	6×10^4^
	*Pediococcus acidilactici* DSM 20284^T^ / AJ305320 (99%–100%)	ND	ND	1.2×10^6^	1.5×10^7^	1.5×10^6^	6×10^5^	2×10^5^	ND	ND
	*Enterococcus faecium* NBRC 100485^T^ / AB681183 (99%–100%)	ND	ND	1×10^6^	1×10^6^	5×10^5^	6×10^5^	9×10^5^	2.1×10^5^	1.7×10^5^
	*Enterococcus mundtii* NBRC 100490^T^ / AB681188 (100%)	ND	ND	ND	ND	1×10^5^	1×10^5^	ND	ND	ND
L-TMR	*Paenibacillus borealis* KK19^T^ / AJ011322 (98%–99%)	3×10^4^	3×10^4^	1.6×10^5^	4×10^3^	ND	ND	ND	ND	ND
	*Paenibacillus turicensis* MOL722^T^ / AF378697 (99%)	4×10^4^	3×10^4^	3×10^4^	ND	ND	ND	ND	ND	ND
	*Paenibacillus xylanexedens* B22a^T^ / EU558281 (99%)	6×10^4^	6×10^4^	1.2×10^5^	1×10^3^	6×10^2^	ND	ND	ND	ND
	*Bacillus pumilus* NBRC 12092^T^ / AB271753 (100%)	2.6×10^3^	2.2×10^3^	1.8×10^3^	6×10^2^	3×10^2^	4×10^2^	2×10^2^	4×10^2^	4×10^2^
	*Bacillus safensis* NBRC 100820^T^ / AB681259 (100%)	2×10^2^	1×10^2^	1×10^2^	2×10^2^	2×10^2^	3×10^2^	2×10^2^	2×10^2^	1×10^2^
	*Bacillus flexus* NBRC 15715^T^ / AB680944 (99%–100%)	3×10^2^	1×10^2^	1×10^2^	1×10^2^	4×10^2^	1×10^2^	1×10^2^	ND	ND
	*Bacillus cereus* NBRC 15305^T^ / AB271745 (99%–100%)	1×10^2^	1×10^2^	1×10^2^	1×10^2^	3×10^2^	1×10^2^	ND	ND	ND
	*Bacillus amyloliquefaciens* NBRC 15535^T^ / AB255669 (99%)	2.2×10^3^	2.7×10^3^	3×10^2^	ND	ND	ND	ND	ND	ND
	*Lactobacillus plantarum* NBRC 15891^T^ / AB326351 (100%)	ND	ND	ND	2.7×10^6^	3.7×10^7^	1.5×10^7^	1.2×10^6^	4.8×10^4^	3.7×10^4^
	*Pediococcus acidilactici* DSM 20284^T^ / AJ305320 (99%–100%)	ND	ND	ND	ND	ND	1×10^7^	9×10^5^	ND	ND
	*Enterococcus faecium* NBRC 100485^T^ / AB681183 (99%–100%)	ND	ND	ND	2.2×10^6^	7×10^6^	1.6×10^7^	5.2×10^6^	2.4×10^4^	3.2×10^4^

The GenBank accession no. of each type strain16S rRNA sequence was shown behind the type strain.

A-TMR, total mixed ration formulated with alfalfa hay; L-TMR, total mixed ration formulated with *Leymus chinensis* hay; FM, fresh matter; ND, not detected.

**Table 5:** Activities and characteristics of proteinases produced by dominant proteolytic bacteria in both TMR silages

Proteolytic bacteria	Accession No.	Proteinase activitiy (U/mL)	Proportions of different classes of proteinase (%)			

			Aspartic proteinase	Serine proteinase	Metallo-proteinase	Cysteine proteinase
*Curtobacterium citreum*	AM410690	3.2–4.7	−	−	+++	−
*Curtobacterium flaccumfaciens*	AJ312209	6.0–6.6	−	−	++	−
*Bacillus amyloliquefaciens*	AB255669	160.2–177.1	−	++	++	−
*Bacillus methylotrophicus*	EU194897	3,24.8–337.5	−	++	++	−
*Bacillus pumilus*	AB271753	40.2–46.8	w	++	+	−
*Bacillus safensis*	AB681259	19.8–27.6	w	++	w	−
*Bacillus flexus*	AB680944	8.4–9.2	w	+	+	−
*Bacillus cereus*	AB598737	2.8–3.5	w	+++	+	−
*Paenibacillus borealis*	AJ011322	5.6–5.8	+	−	++	−
*Paenibacillus xylanexedens*	EU558281	9.2–9.6	w	−	+	−
*Paenibacillus turicensis*	AF378697	5.7–6.4	w	−	+	−
*Lactobacillus plantarum*	AB326351	1.0–1.4	−	+++	+	−
*Enterococcus faecium*	AB681183	2.8–4.0	−	++	+++	−
*Enterococcus mundtii*	AB681188	12.0–14.4	−	++	+++	−
*Pediococcus acidilactici*	AJ305320	1.6–2.8	−	w	++	−

−, proteinase activity was not suppressed by the inhibitor; w, activity was suppressed by <25%; +, activity was suppressed by 25% to 50%; ++, activity was suppressed by 50% to 75%; +++, activity was suppressed by 75% to 100%.
